# Metagenomic analyses and genetic diversity of *Tomato leaf curl Arusha virus* affecting tomato plants in Kenya

**DOI:** 10.1186/s12985-020-01466-z

**Published:** 2021-01-06

**Authors:** Edith Khamonya Avedi, Adedapo Olutola Adediji, Dora Chao Kilalo, Florence Mmogi Olubayo, Isaac Macharia, Elijah Miinda Ateka, Eunice Magoma Machuka, Josiah Musembi Mutuku

**Affiliations:** 1 Department of Phytosanitary Services and Biosafety, Kenya Plant Health Inspectorate Service, Nairobi, Kenya; 2 Department of Plant Science and Crop Protection, University of Nairobi, Nairobi, Kenya; 3 Biosciences Eastern and Central Africa, International Livestock Research Institute, Nairobi, Kenya; 4 Department of Crop Protection and Environmental Biology, Faculty of Agriculture, University of Ibadan, Ibadan, Nigeria; 5 Department of Horticulture and Food Security, Jomo Kenyatta University of Agriculture and Technology, Nairobi, Kenya; 6 Department of Plant Sciences, University of Cambridge, Cambridge, CB2 3EA UK; 7 Present Address: The Central and West African Virus Epidemiology (WAVE), Pôle Scientifique et d’Innovation de Bingerville, Université Félix Houphouët-Boigny, Abidjan, Côte d’Ivoire

**Keywords:** *Solanum lycopersicum*, Begomovirus, Phylogeny, Haplotype diversity, Tajima’s D

## Abstract

**Background:**

Tomato production is threatened worldwide by the occurrence of begomoviruses which are associated with tomato leaf curl diseases. There is little information on the molecular properties of tomato begomoviruses in Kenya, hence we investigated the population and genetic diversity of begomoviruses associated with tomato leaf curl in Kenya.

**Methods:**

Tomato leaf samples with virus-like symptoms were obtained from farmers’ field across the country in 2018 and Illumina sequencing undertaken to determine the genetic diversity of associated begomoviruses. Additionally, the occurrence of selection pressure and recombinant isolates within the population were also evaluated.

**Results:**

Twelve complete begomovirus genomes were obtained from our samples with an average coverage of 99.9%. The sequences showed 95.7–99.7% identity among each other and 95.9–98.9% similarities with a *Tomato leaf curl virus Arusha virus* (ToLCArV) isolate from Tanzania. Analysis of amino acid sequences showed the highest identities in the regions coding for the coat protein gene (98.5–100%) within the isolates, and 97.1–100% identity with the C4 gene of ToLCArV. Phylogenetic algorithms clustered all Kenyan isolates in the same clades with ToLCArV, thus confirming the isolates to be a variant of the virus. There was no evidence of recombination within our isolates. Estimation of selection pressure within the virus population revealed the occurrence of negative or purifying selection in five out of the six coding regions of the sequences.

**Conclusions:**

The begomovirus associated with tomato leaf curl diseases of tomato in Kenya is a variant of ToLCArV, possibly originating from Tanzania. There is low genetic diversity within the virus population and this information is useful in the development of appropriate management strategies for the disease in the country.

## Background

Tomato (*Solanum lycopersicum*) is an important vegetable grown worldwide for its commercial and high nutritional value [1, 2]. Tomato fruits are rich in ascorbic acid, retinol and lycopene with antioxidant properties that fight cancer [3]. In Kenya, annual tomato production is approximately 410,033 tons, valued at Ksh. 14 billion [4, 5]. The crop is a major source of income for smallholder rural farmers and it is produced predominantly for the domestic market [6]. The main producing counties are Kirinyaga, Kajiado, Nakuru, Meru, Bungoma and Taita Taveta. Despite the intensified production of tomato in Kenya, yields from tomato farms are low due to biotic and abiotic constraints [7]. Biotic constraints include insect pests and diseases caused by various bacteria, fungi, nematodes and viruses [7]. Although diseases caused by bacteria, fungi and nematodes cause significant yield losses in tomato production, the effect of virus infections on production has been given relatively low attention.

Virus diseases are considered as the third significant constraint to tomato production [8]. There are about 136 viruses that infect tomato [9] of which 60 belong to the genus *Begomovirus* and family Geminiviridae [10]. Begomoviruses are transmitted by whitefly (*Bemisia tabaci* Gennadius) in a persistent manner, leading to yield losses of up to 100% in tropical and subtropical regions [11]. They possess circular single-stranded DNA (ssDNA) genomes, classified as either mono- or bipartite [12]. Bipartite begomoviruses possess two ssDNA molecules, identified as DNA-A and –B whereas, monopartite begomoviruses have only DNA-A which is capable of solely inducing diseases [13]. Most begomoviruses from the Old World (mainly Africa and Asia) are monopartite and possess satellites known as alpha-, beta- or delta- satellites [14]. The genomes of monopartite begomoviruses are ~ 2.8 kb in size with genes in both directions that diverge from a non-coding intergenic region (IR). The region has promoter elements including the *ori* of virion-strand DNA replication [15]. The DNA-A component of begomoviruses contains five or six open reading frames (ORFs) that encode ~ 10 kDa proteins [16]. These proteins play various roles in virus assembly, virus replication, host gene regulation, silencing suppression and vector transmission [11]. Like most plant viruses, begomoviruses evolve rapidly through recurrent mutations and recombination events, leading to the emergence of novel pathotypes that exploit new environments and challenge host resistance [17, 18]. Natural occurrences of recombinants are known to lead to emergence of more virulent viruses or novel strains with new hosts and properties [18].

The leaf curl disease of tomato, caused by several begomoviruses, is a widespread threat to tomato production in many tropical and subtropical regions worldwide [9, 19]. Symptoms include yellowing of upper leaves, excessive branching, reduced leaf sizes, puckering of leaves, curling upwards of margins, stunting and flower abscission [9]. In Kenya, the disease symptoms were first observed in 1997 across tomato fields and *Tomato yellow leaf curl virus* (TYLCV) was identified as its causative agent [19]. However, there has been no effort afterwards to characterize the virus populations. Several approaches are available for begomovirus identification, ranging from serological techniques to deep sequencing approaches [20]. Since begomoviruses species and strains cause diseases with similar symptoms in tomato, the use of serological assays has limitations as antibodies are able to cross-react with closely-related viruses or virus strains, thus making strain identification difficult. Recent advances in sequencing technologies have provided better approaches for identification and characterization of plant viruses in Kenya [21–24].

Metagenomics is the analysis of microbial and virus populations in environmental samples through nucleic acid sequencing methods [25]. Motivations for performing plant virus metagenomics include the identification of causal organisms associated with virus diseases in crops, screening for specific viruses when their presence is suspected, detection of asymptomatic or cryptic viruses and the discovery of novel viruses among other microorganisms [22]. In this study, a metagenomics approach was used to identify the viruses associated with leaf curl within tomato plants from farmers’ fields in Kenya. The virus populations were further evaluated for their genetic diversity, evidence of recombination and occurrence of selection pressure.

## Methods

### Sample collection and extraction of nucleic acids

**Fig. 1:**
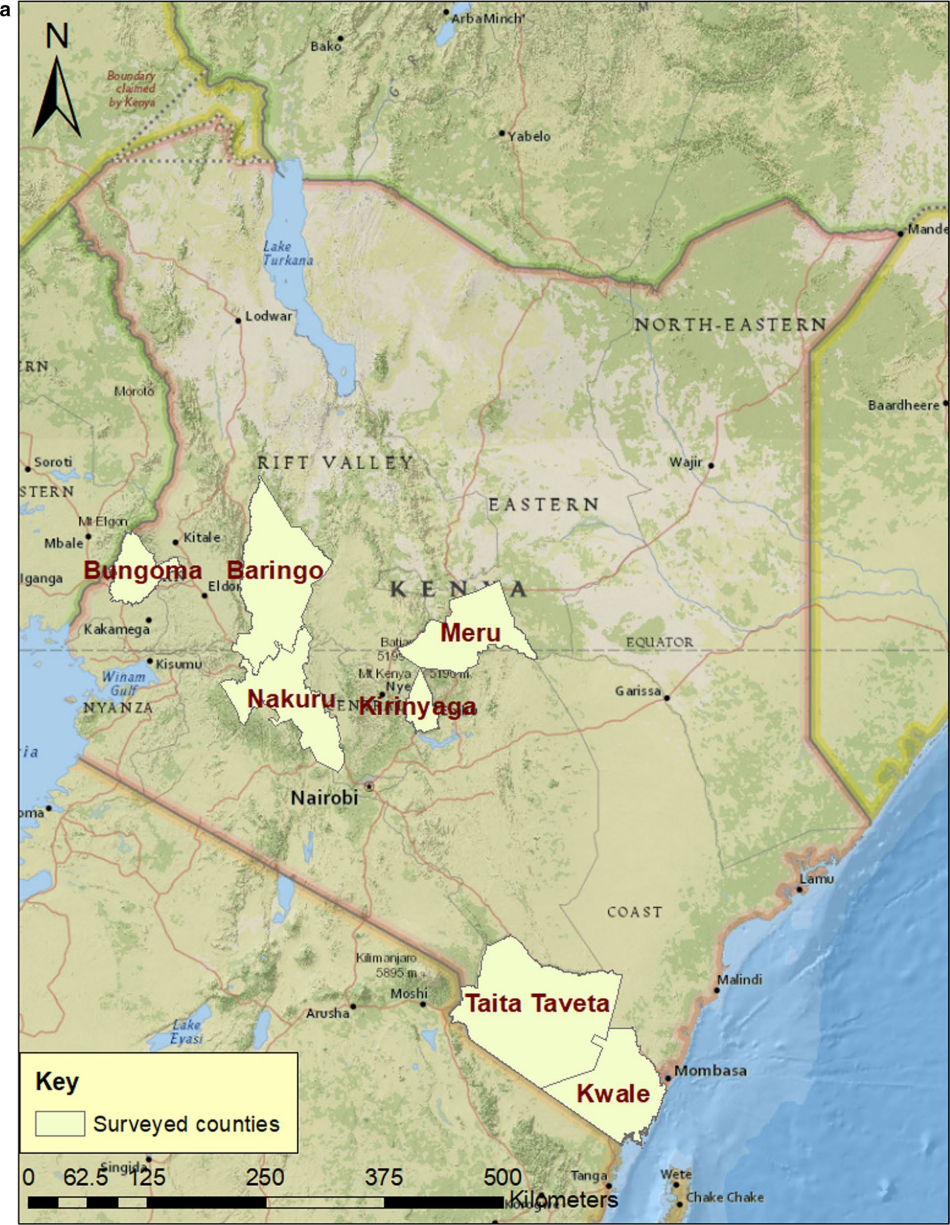

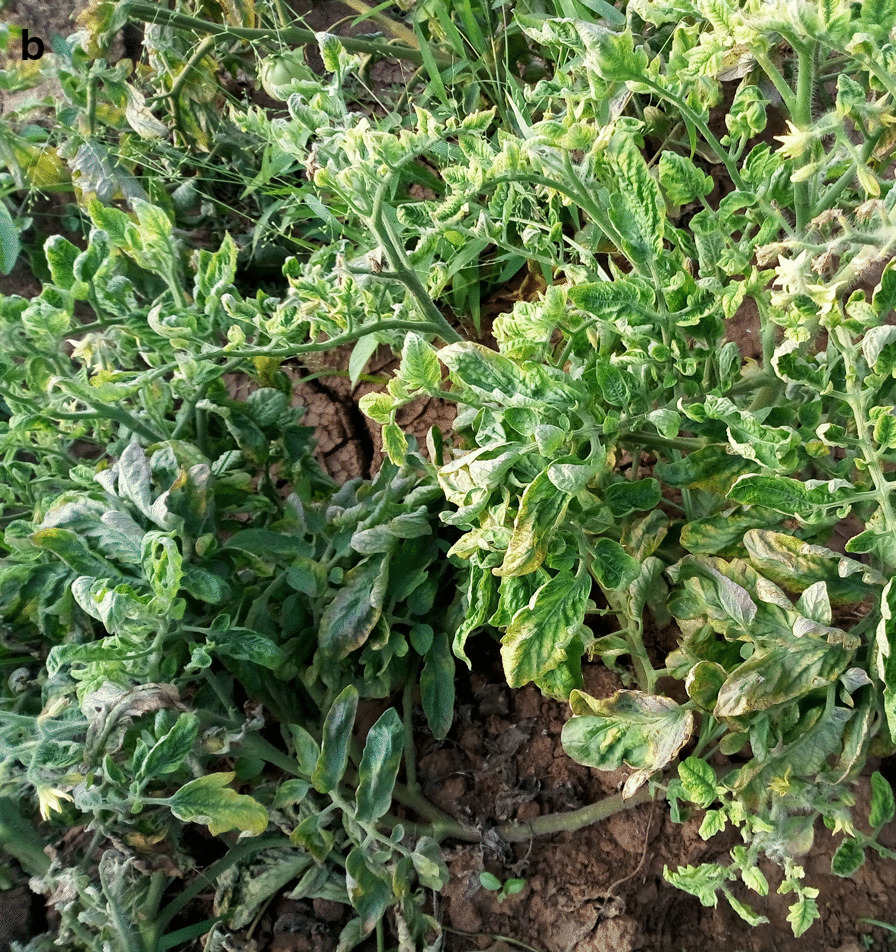
**a** The map of Kenya showing counties where tomato fields were sampled for this study. **b** Photograph of symptomatic tomato plants showing leaf curl from farmer fields

Field surveys and sampling were carried out between January and May 2018 in four major tomato growing regions in Kenya, with different agro-ecological and climatic conditions (Fig. 1a). Tomato fields were randomly selected based on crop availability, with 30 plants randomly assessed per field. From each field, young trifoliate leaf samples (n = 5) were obtained only from plants showing symptoms such as chlorosis, reduced leaf size, upward leaf curling, stunting and flower abscission (Fig. 1b). A total of 240 leaf samples were collected from 48 fields, carried in paper bags and stored at − 80 °C till further analysis. Samples from each field were pooled prior to DNA extraction.

Extraction of total genomic DNA was performed as described [26]. Briefly, about 150 mg of leaf tissues were homogenized using a mortar and pestle with 1.5 ml of pre-warmed extraction buffer (2% cetyl trimethyl ammonium bromide w/v; 100 mM Tris–HCl, 1.4 M NaCl, 20 mM EDTA, pH 8.0 + 50 mg PVP + 0.2% v/v β –mercaptoethanol added just before use). The samples were transferred into 1.5 ml microtubes and incubated at 65 °C for 30 min while mixing at 10 min interval. The tubes were centrifuged at 10,000 rpm for 5 secs and supernatants (750 µl) were transferred into fresh microtubes. Chloroform and isoamyl alcohol (750 µl) in the ratio 24:1 was added to the tubes, mixed and centrifuged at 10,000 rpm for 15 min. The aqueous layers were transferred into new microtubes and ice cold isopropanol (300 µl) were added and mixed by inverting the tube slowly. Tubes were incubated overnight at − 20 °C and the nucleic acids were then pelleted by centrifugation at 10,000 rpm for 15 min. The supernatants were discarded, pellets washed with 500 µl of 70% (v/v) ethanol and dried at room temperature. These were dissolved in 100 µL of Tris–EDTA buffer (10 mM Tris–HCl [pH 8.0] + 1 mM EDTA), incubated at 37 °C for 30 min and stored at − 20 °C. A Nanodrop 2000 spectrophotometer (Thermo Fisher Scientific, MA, USA) was used to determine the quality of the nucleic acids.

### Library preparation and sequencing

The genomic DNA were quantified using a Qubit™ fluorometer (Thermo Fisher Scientific, MA, USA) and normalized to 2.5 ng/µl and used for library preparation. Libraries were prepared using Nextera DNA library preparation kit (Illumina, CA, USA) according to the manufacturer’s instructions. Briefly, enzymatic fragmentation was carried out on normalized genomic DNA samples (20 µl) via addition of TD buffer (25 µl) and TDE (5 µl). Mixtures were centrifuged (Hettich Centrifugen, D-78532, Germany) at 14,000 rpm at 20 °C for 1 min and transferred into microtubes. Tagmentation was carried out in a pre-programmed thermocycler at 55 °C lid and 55 °C incubation temperature, while holding at 10 °C. The tagmented DNA was barcoded using indexed adapters then cleaned with AMPure XP magnetic beads (Beckman Coulter, Inc. Indianapolis, IN) to remove shorter DNA fragments and other impurities. Library quality was confirmed with the Agilent Tape Station 2200 System (Agilent Technologies, Santa Clara, CA). All the 48 libraries were quantified using the Qubit™ fluorometer (Thermo Fisher Scientific Inc., Waltham, MA). The indexed DNA libraries of 48 biological samples were each normalized to a concentration of 4 nm before being pooled together. High-throughput sequencing was performed on an Illumina MiSeq System using 2 × 251 v2 kit and 12 pM of 1% PhiX v3 spike to create paired-end reads. Sequencing was performed at the facility of the Biosciences Eastern and Central Africa International Livestock Research Institute (BecA-ILRI) Hub, Nairobi, Kenya.

### Sequence processing and assembly

After sequencing, quality control of fastq paired end reads was performed using FastP v.0.20.0 [27] to remove adapters, poly-N sequences (≥ 15%) and filter off low quality reads. High-quality reads were then mapped to the tomato genome (GenBank RefSeq accession number GCA_000188115.3) using Bowtie v.2.3.4.3 [28] under default parameters. Unmapped reads were assembled into contigs de novo using MEGAHIT v.1.1.3 [29] with default settings and those representing ssDNA sequences were verified using Kaiju virus database [30]. The sequences were then subjected to BLASTN 2.9.0 + [31] to determine similarity match and virus identification (Additional file 1: Fig. S1). Protein prediction of ORFs was determined using ORF Finder (http://www.ncbi.nlm.nih.gov/projects/gorf).

### Sequence validation through polymerase chain reaction (PCR) and Sanger sequencing

The assembled begomovirus genomes were validated using a polymerase chain reaction (PCR) step followed by Sanger sequencing of the amplified products. The Illumina assembled virus sequences were aligned together using ClustalW multiple sequence alignment program with default parameters as implemented in BioEdit v.7.2.3 [32]. A consensus sequence was obtained and used to design PCR primers ToLCV-Forward (5′-ATTGGCGATTTCCCAGGTATAG-3′) and ToLCV-Reverse (5′-ACAATGTGGGCTAGGTCATTAG-3′) using the Primer Express v3.0 software (Applied Biosystems, USA). Secondary structures, complementarity and dimer effects of the primers were also checked using the multiple primer analyzer software (Thermo Fisher Scientific, MA, USA). Using PCR, these were tested on the genomic DNA from which the complete begomovirus genomes had been obtained via Illumina sequencing. The PCR products were ethanol-purified and quantified using a Nanodrop 2000 spectrophotometer (Thermo Fisher Scientific, MA, USA) to determine purity levels. Amplicons were sequenced at Macrogen Europe and manually assembled using BioEdit. Consensus sequences were verified using BLASTN 2.9.0 + and comparisons were made with the complete begomovirus sequences assembled from Illumina reads.

### Sequence alignment, distance matrix and evidence of recombination

Complete sequences of monopartite begomoviruses found in tomato were retrieved from GenBank (Additional file 2: Table S1) and aligned with full virus contigs using ClustalW in BioEdit. Deduced amino acids from the ToLCV genomes were compared with GenBank isolates while sequence pairwise identities were performed using SDT v1.2 [33] with pairwise gap deletions. A scan for recombination signatures were performed on each protein-coding sequence data using the single breakpoint scanning (SBP) and genetic algorithm recombination detection (GARD) methods [34]. These two methods were implemented by the Datamonkey software [35]. Potential recombination events were further investigated using the default settings of the seven detection algorithms within RDP v 4.13 [36]. Putative recombination events, potential recombinants, and their parental sequences were deemed acceptable only when signals were identified by at least four detection methods, with strong levels of significance (*P* ≤ 0.05).

### Phylogeny, genetic diversity and population genetic analysis

A phylogenetic tree was constructed using the maximum likelihood method based on Jukes-Cantor model in MEGA v.6.06 [37]. Bootstrap replicate values were set at 1,000 while a strain of *Tomato leaf curl purple vein virus* (KY196216) was selected as an outgroup. Genetic structure and diversity within ToLCV populations in Kenya were investigated to understand potential evolutionary dynamics that produce variations. Population structure parameters estimated included; average nucleotide diversity (π), haplotype diversity (Hd), number of polymorphic or segregating sites (S), the statistic estimate of population mutation based on the number of segregating sites (θ-W), total number of mutations (Eta), the average number of nucleotide differences between sequences (k) and the statistic estimate of population mutation based on the total number of mutations (θ-Eta). These were estimated using complete genome and protein coding sequences in DnaSP v5.10.01 [38].

The possible occurrences of selection pressure on individual genes and sites within the ToLCV populations were obtained using the single-likelihood ancestor counting (SLAC) method [39] in the HyPhy package [40] as implemented on the Datamonkey software [35] at http://www.datamonkey.org. The ratio of average number of nucleotide differences between the sequences per nonsynonymous site (d_N_) to the average number of nucleotide differences between the sequences per synonymous site (d_S_) were calculated as an indicator of natural selection. These were used to estimate the occurrence of positive and negative selection at typical begomovirus amino acid ORF sites: the movement protein (MP) or V1 protein, coat protein (CP) or V2 protein, replication protein (Rep) or C1 protein, transcription activator protein (TrAP) or C2 protein, Rep enhancer protein (REn) or C3 protein and the C4 protein. Depending on the dN/dS values, the selection pressure was considered negative or purifying (dN/dS < 1), neutral (dN/dS = 1), or diversifying or positive (dN/dS > 1) for data sets of each coding region. The DNAsp v5.10.01 was used to calculate the Tajima's D, Fu and Li's F* and D*, and Fu's Fs to determine the deviation of ToLCV populations from neutrality assuming a constant population size, with zero recombination and migration [41]. A negative Tajima’s D statistic indicates superfluous low-frequency polymorphism triggered by background selection, genetic hitchhiking, or population expansions [42]. Conversely, positive values of Tajima’s D statistic suggest minimal levels of low and high frequency polymorphisms, indicating a reduction in population size and/or balancing selection.

## Results

### Sequence data, de novo assembly and begomovirus PCR verification

After mapping of sequence reads from leaf samples to the tomato reference genome, unmapped reads were subsequently assembled into contigs. The de novo assembly yielded several contigs, with the largest having sizes of > 45 kb while N50 values ranged from 135–270 bp (Additional file 3: Table S2). After Kaiju analyses (see Materials and Methods), all assembled virus contigs were subjected to BLASTN 2.9.0 + searches. The results revealed twelve contig matches of lengths > 2.7 kb from eleven samples with complete begomovirus genomes within the database (see Additional file 2: Table S1) while partial contigs matching other DNA viruses were also present (data not shown). Raw reads from these positive samples have been deposited at the SRA archive (Bioproject number PRJNA646848). Across all the samples, only monopartite begomoviruses with DNA-A-like sequences were recovered. The presence of beta-satellites was not evaluated in this study. However, a sample (Tom54) produced the full-length genome of a separate begomovirus, *Chickpea chlorotic dwarf virus*, which we recently described [43]. The PCR primers designed from the full begomovirus genomes produced the expected 530 bp amplicons from the genomic DNA of infected tomato plants. Sanger sequencing of the PCR products revealing 95.6–99.7% identity (data not shown) with the complete genomes assembled from the Illumina reads, thus confirming the accuracy of the nucleotides within the assembled virus genomes.

### The begomoviruses in Kenyan tomato are a variant of ToLCArV

In all the samples, the full-length genomes of the begomoviruses varied from 2760 to 2765 bp (Table 1). These were subsequently deposited in GenBank database under the accession numbers MN894493 to MN894504. Sequence analyses showed that these genomes encoded the six ORFs (V1, V2, C1, C2, C3 and C4) that are typical of monopartite begomoviruses while the intergenic regions ranged from 245–250 nt. Pairwise alignments of begomoviruses (see Additional file 4: File 1) with pairwise deletion of gaps revealed the highest full genome similarity (95.9–98.9%) with an isolate of *Tomato leaf curl Arusha virus* (ToLCArV, GenBank accession EF194760) from Tanzania (Additional file 5: Table S3). This was followed by *Tomato leaf curl Toliara virus* (ToLCToV, GenBank accession AM701768) with 95.9–98.9% identity and another isolate of *Tomato leaf curl virus Arusha virus* (ToLCArV, GenBank accession DQ519575) at 89.8–90.5% similarity. Furthermore, all isolates exhibited less than 80% pairwise sequence identity to other begomovirus sequences (Additional file 6: Fig. S2). Based on the species demarcation criteria of the International Committee for the Taxonomy of Viruses set for begomoviruses at < 91% nucleotide sequence identity [44], the Kenyan begomoviruses were considered as a variant of ToLCArV. Similar patterns were observed for deduced amino acids as the highest identity was observed with ToLCArV (GenBank accession EF194760) across all the six coding regions (93.3–99.1 for MP, 97.3–98.9% for CP, 95.4–98.6% for Rep, 94.2–97.8% for TrAP, 96.0–98.0% for REn and 97.1–100% for C4 protein). Pairwise comparison across amino acids of other tomato infecting monopartite begomoviruses revealed similar patterns (Additional file 7: Table S4). Further analyses revealed 95.7–99.7% similarity within the twelve Kenyan ToLCArV-like isolates while amino acid residues also revealed high similarities at the MP (94.1–100%), CP (98.5–100%), Rep (94.1–99.4%), TrAP (94.3–100%), REn (95.6–100%) and C4 (95.1–100%) coding regions (Table 2).

**Table 1:** Summary of virus identification of contigs from tomato samples in Kenya by BLAST and their identity with closest database homologues

Sample	Length of virus contigs (nt)	Virus identified	Accession number	Similarity (%)	Query cover (%)	Identities	E-value
Tom 5b	2761	*Tomato leaf curl Arusha virus*	EF194760	97.72	99	2698/2761	0
Tom 5b	2765	*Tomato leaf curl Arusha virus*	EF194760	97.69	100	2701/2765	0
Tom 46	2763	*Tomato leaf curl Arusha virus*	EF194760	96.16	100	2658/2764	0
Tom 13	2762	*Tomato leaf curl Arusha virus*	EF194760	95.84	100	2648/2763	0
Tom 14	2760	*Tomato leaf curl Arusha virus*	EF194760	98.81	100	2729/2762	0
Tom 45	2763	*Tomato leaf curl Arusha virus*	EF194760	98.84	100	2731/2763	0
Tom 39	2762	*Tomato leaf curl Arusha virus*	EF194760	97.10	100	2683/2763	0
Tom 27	2762	*Tomato leaf curl Arusha virus*	EF194760	98.91	100	2732/2762	0
Tom 35	2762	*Tomato leaf curl Arusha virus*	EF194760	96.45	100	2665/2763	0
Tom 28	2763	*Tomato leaf curl Arusha virus*	EF194760	95.98	100	2653/2764	0
Tom 37	2762	*Tomato leaf curl Arusha virus*	EF194760	95.91	100	2651/2764	0
Tom 22	2761	*Tomato leaf curl Arusha virus*	EF194760	96.60	100	2668/2762	0

**Table 2:** Percentage pairwise sequence identities among the twelve *Tomato leaf curl virus Arusha virus*-like isolates from Kenya

Segment^a^	Nucleotide (%)	Amino acid (%)
Genome	95.7–99.7	–
V1	95.0–100	94.1–100
V2	95.0–100	98.5–100
C1	95.7–99.6	94.1–99.4
C2	95.0–100	94.3–100
C3	96.8–100	95.6–100
C4	98.7–100	95.1–100

^a^V1: Movement protein gene, V2: Coat protein gene, C1: Replication-associated protein gene, C2: Transcriptional activator protein gene, C3: Replication enhancer protein gene, C4: C4 protein gene

### Recombination analyses

Using the automated SBP and GARD tools within Datamonkey, recombination signals were found within the genomic regions of our ToLCArV-like populations (data not shown). However, further analyses of the isolates (see Additional file 8: File 2) using the programs implemented in the RDP4 software did not reveal significant recombination signals within our sequences. Conversely, two isolates Tom5a (MN894493) and Tom39 (MN894499) were identified as potential major and minor parental sequences for the signals detected in ToLCArV (DQ519575) and ToLCDiV (AM701765), respectively (Table 3).

**Table 3:** Identification of Kenyan *Tomato leaf curl virus Arusha virus*-like isolates as parents of putative recombinant tomato begomoviruses using the RDP4 software

Recombinants^a^	Potential parents^b^		Recombination breakpoints	Average *p* values in detecting algorithms^c^						
	Minor	Major		R	G	B	M	C	S	T
ToLCArV	ToLCUV	ToLCArV	158–524	2.42E−06	2.04E−04	–	7.13E−09	4.67E−10	–	1.68E−12
TZTen05-Tanzania (DQ519575)	Iganga-Uganda (DQ127170)	Tom5a-Kenya (MN894493)								
ToLCDiV	ToLCArV	ToLCMohV	1091–1583	1.48E−12	4.75E−12	1.32 E−10	1.04E−10	1.17E−05	–	4.29E−04
Namakely-Madagascar (AM701765)	Tom39-Kenya (MN894499)	Moheli-Comoros (AM701763)								

^a^ToLCArV: Tomato leaf curl virus Arusha virus, ToLCDiV: Tomato leaf curl Diana virus

^b^ToLCUV: ToLCUV: Tomato leaf curl Uganda virus, ToLCMohV: Tomato leaf curl Moheli virus

^c^R: RDP, G: GENECONV, B: BootScan, M: MaxChi, C: Chimaera, S: SiScan, T: 3Seq

### Phylogenetic relationships and genetic diversity of Kenyan tomato begomoviruses

**Fig. 2:**
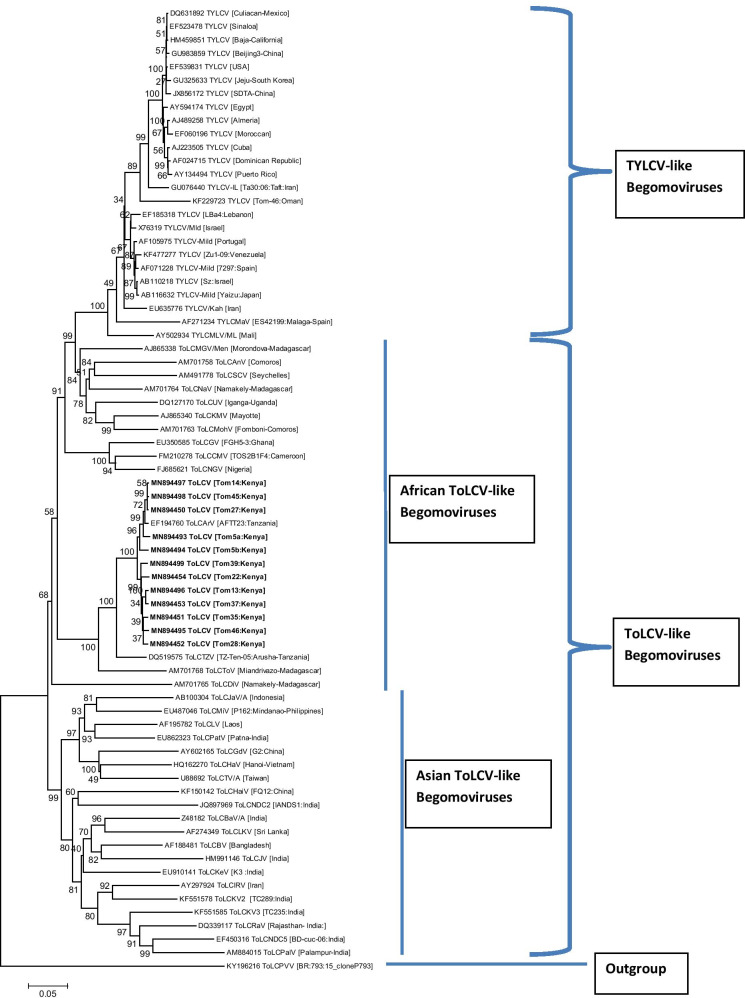
Phylogenetic analyses of tomato leaf curl virus from Kenya (n = 12) with selected worldwide begomoviruses based on alignment of complete DNA-A nucleotide sequences. The tree was generated using the maximum likelihood inference based on the Jukes-Cantor model as implemented in MEGA v.6.06 [37]. Percentage bootstrap support values (1,000 iterations) are indicated at the branch nodes. The tree is rooted with *Tomato leaf curl purple vein virus* (accession number KY196216) as an outgroup. The scale bar shows the number of nucleotide substitutions power site. Details of all the isolates are provided in Additional file 2: Table S1

A phylogenetic analysis was done using the full genome sequences of the 12 ToLCArV isolates from Kenya, together with TYLCV-like sequences and other tomato begomoviruses from GenBank. As expected, all TYLCV-like isolates (n = 25) clustered separately from ToLCV-like sequences (n = 46) with a clear geographical segregation (Fig. 2). African ToLCV-like sequences (n = 26) were separated from those of Asian origins (n = 20) while isolates from Kenya formed a monophyletic cluster with isolates from Tanzania (ToLCArV, EF194760 and DQ519575) (Fig. 2). This finding strengthens the hypothesis that Kenyan ToLCArV-like isolates are closely related to ToLCArV from Tanzania, with both strains having a common ancestor.

Analyses of haplotype number and haplotype diversity, represented by ‘h’ and ‘Hd’, respectively revealed varying values among the 12 Kenyan ToLCArV-like sequences and also among other ToLCV-like sequences from GenBank, based on the six coding regions evaluated (Table 4). From the total ToLCV-like sequences (n = 46), haplotypes number ranged from 43 in the MP region to 46, in the CP, Rep and whole genomes. Similarly, among the Kenyan ToLCArV-like isolates (n = 12), ‘h’ values ranged from 9 (MP gene) to 12 (CP, Rep and complete genomes). Thus, across ToLCV-like sequences from the Genbank and the Kenyan ToLCArV-like sequences, each isolate represented a haplotype at both CP and Rep genes, revealing high genetic variation within the coding regions of each group. This therefore indicates that genetic variation was highest within the CP and Rep coding regions. Interestingly, Hd values were highest for the CP and REn gene and lowest for MP gene, both across ToLCV-like isolates obtained from GenBank and among the 12 Kenyan ToLCV-like sequences obtained in this study (Table 4). Furthermore, genetic distances for each gene-specific data set were calculated, with highest π values obtained within the REn gene (0.2458) across the ToLCV-like isolates (n = 46). The C4 gene and Rep gene recorded the lowest π values i.e. 0.21015 and 0.21165, respectively. Remarkably, the π value of the C4 gene within the 12 Kenyan ToLCArV-like isolates (0.00869) was more than half the π values of other coding regions, indicating that these coding regions were more variable than the C4 gene (Table 4). Collectively, these results show high genetic variability among the CP and Rep coding regions across both ToLCV groups, with C4 gene having the least variation across the isolates.

**Table 4:** Genetic variability determinants and neutrality tests on *Tomato leaf curl virus Arusha virus*-like populations from Kenya with other worldwide tomato begomoviruses

Population	Gene^a^	N^b^	h^c^	S^d^	Hd^e^	Eta^f^	*π*^g^	k^h^	θ-W^i^	θ-Eta^j^	Tajima's *D*	Fu and Li's *D*	Fu and Li's *F*
Tomato begomoviruses	Genome	2920	46	1666	1.000	2985	0.22424	569.5749	0.14924	0.2674	− 0.5971	− 0.2912	− 0.4859
(n = 46)	V1	372	43	241	0.996	419	0.22538	76.1768	0.16224	0.28206	− 0.7389	− 0.8229	− 0.9463
	V2	1261	46	752	1.000	1334	0.22421	271.971	0.14106	0.25023	− 0.3842	− 0.0052	− 0.1764
	C1	1179	46	692	1.000	1213	0.21165	225.4106	0.14784	0.25915	− 0.6771	− 0.3609	− 0.5737
	C2	431	45	264	0.999	458	0.23777	97.486	0.14651	0.25417	− 0.2374	− 0.0923	− 0.1752
	C3	469	45	303	0.999	550	0.24589	108.1903	0.15669	0.28442	− 0.4989	− 0.3631	− 0.4949
	C4	316	44	210	0.997	360	0.21015	65.1459	0.15414	0.26423	− 0.7516	− 0.4512	− 0.6736
Kenyan ToLCArV-like	Genome	2766	12	211	1.000	224	0.0264	72.955	0.0253	0.0268	− 0.0774	− 0.4189	− 0.3751
isolates (n = 12)	V1	360	9	22	0.939	24	0.02298	8.2727	0.02024	0.02208	0.1819	0.1216	0.1564
	V2	1239	12	71	1.000	72	0.0209	25.8788	0.01899	0.01926	0.3958	− 0.0631	0.0659
	C1	1116	12	94	1.000	102	0.02744	30.6212	0.02789	0.03027	− 0.4355	− 0.7775	− 0.7841
	C2	422	11	39	0.985	40	0.02761	11.6515	0.0306	0.03139	− 0.5481	− 1.072	− 1.065
	C3	450	11	23	0.985	23	0.01872	8.4242	0.01692	0.01692	0.4703	− 0.3193	− 0.1286
	C4	312	10	11	0.955	11	0.00869	2.7121	0.01167	0.01167	− 1.0628	− 1.4718	− 1.552

^a^V1: Movement protein gene, V2: Coat protein gene, C1: Replication-associated protein gene, C2: Transcriptional activator protein gene, C3: Replication enhancer protein gene, C4: C4 protein gene

^b^N: Number of nucleotide sites

^c^h: Haplotype number

^d^S: Total number of variable or segregation sites

^e^Hd: Haplotype diversity

^f^Eta: Total number of mutations

^g^π: Nucleotide diversity

^h^k: Average number of nucleotide differences between sequences

^i^θ-W: Waterson’s estimate of population mutation rate based on the total number of segregating sites

^j^θ-Eta: Waterson’s estimate of population mutation rate based on the total number of mutations

### Tajima’s D and estimation of selection pressure

Tajima’s D statistical test [45] was used to evaluate the nucleotide polymorphism occurring within each gene and on the complete genomes of Kenyan ToLCArV-like isolates and other ToLCV-like isolates. The Tajima’s D, Fu and Li's D and Fu and Li's F statistic revealed negative values for the complete genome datasets which did not statistically deviate from zero (*P* > 0.10) (Table 5). Within Kenyan isolates, similar trends were observed for gene-specific datasets except the MP and CP genes which revealed positive values that are not significantly (*P* > 0.10) different. These results indicate an excess of low-frequency polymorphism caused by background selection, genetic hitchhiking, or population increases.

**Table 5:** Estimates of selection pressure on the coding regions of twelve *Tomato leaf curl virus Arusha virus* -like isolates from Kenya

Coding region^a^	Total number of sites	*log* L	d_N_	d_S_	d_N_/d_S_	Number of sites^b^	
						Positive or diversifying selection	Negative or purifying selection
V1	114	− 651.20	0.0363	0.1757	0.2067	0	1
V2	258	− 1483.95	0.0150	0.2212	0.0677	0	3
C1	364	− 2392.81	0.0847	0.2124	0.3986	0	11
C2	135	− 856.05	0.0607	0.2085	0.2908	0	1
C3	134	− 708.30	0.0307	0.1184	0.2590	0	2
C4	85	− 509.31	0.0392	0.0341	1.1491	0	0

^a^V1: Movement protein gene, V2: Coat protein gene, C1: Replication-associated protein gene, C2: Transcriptional activator protein gene, C3: Replication enhancer protein gene, C4: C4 protein gene

^b^Selected at *P* ≤ 0.1

In order to understand the selection pressure acting on the different coding regions within our ToLCArV-like sequences, the ratios of nonsynonymous substitution per nonsynonymous site (dN) and synonymous substitutions per synonymous sites (dS) were calculated (Table 5). The dN/dS ratio is an estimator of the evolutionary constraints imposed on a coding region with a value > 1 considered as evidence for positive selection, values < 1 show evidence of negative selection while values of 1 indicate neutral selection [46]. Across the Kenyan ToLCArV-like sequences, the dN/dS ratio was 0.2067 for the MP gene, 0.067 for the CP gene, 0.3986 for Rep gen, 0.2590 for REn Gene, 0.2908 for TrAP gene and 1.1491 for C4 gene (Table 5). Thus, contrasting patterns of evolution were obtained for the coding region datasets as all except the C4 gene had dN/dS ratio of < 1. This indicates a negative or purifying selection among five out of six coding regions. In addition, these results show that although the MP, CP, Rep, TrAP and REn coding regions are under strong purifying selection, the purifying selective pressure is not distributed uniformly across the genes. The protein encoded by the C4 gene appears to be selectively neutral. The dN/dS values for the CP gene had the lowest values, with other gene sets having at least more than thrice its dN/dS ratio (Table 5).

## Discussion

Tomato production in Kenya is widespread and has been limited by the impact of the tomato leaf curl disease. *Tomato yellow leaf curl virus* has always been assumed to be the causal because of the typical yellow leaf curl symptoms commonly associated with tomato in Africa. Indeed, a tomato leaf curl-like virus infecting tomato in Kenya has previously been reported [47]. The paucity of information on viruses of high economic importance is compounded by the fact that only a few studies from Kenya have described the genomic properties of begomoviruses from cassava [48], sweet potato [49] and a non-cultivated weed host [50]. Using a metagenomics approach, we have described the occurrence of monopartite begomoviruses associated with the leaf curl disease of tomato in Kenya. Our results show that a genetically distinct begomovirus is associated with the disease in Kenya. Analyses of the complete genomes and coding regions of these begomoviruses, together with the failure to detect the presence of DNA-B component affirms that these virus populations were members of the Old World monopartite begomovirus species. Our findings represent the first comprehensive description of full begomovirus genomes from tomato in Kenya. This information is crucial for understanding the causal agents associated with the tomato leaf curl disease and its properties as a first step towards appropriate robust disease management. The availability of full genome sequences will help to elucidate further the evolutionary behavior of the virus.

All the Kenyan ToLArV-like sequences obtained in this study, shared very high nucleotide and amino acid sequence similarities, indicating low intra-population genetic diversity. Similar conclusions have been reached in other studies on tomato begomoviruses [51, 52]. Curiously, we observed that the nucleotide sequences of the 12 ToLCArV-like isolates shared high identities among themselves but shared lower sequence identities with other begomoviruses. This is likely as a result of the genetic bottleneck imposed through the method of begomovirus transmission by whiteflies [53]. Our study did not investigate virus occurrence within vectors. Nevertheless, the high genetic similarity within the population in our result could be due to a ‘founder effect’ arising from ecological and epidemiological factors such as vector or seed-mediated spread possibly from Tanzania. The derived amino acid sequences of the population in our results show homologous characteristic with other monopartite begomoviruses, indicating possible similar biological behaviors.

Results from sequence similarity indices, together with phylogenetic inferences, suggest that the ToLCArV-isolates associated with tomato leaf curl diseases in Kenya were likely of Tanzanian origin. The homogeneity of nucleotide and amino acids as well as phylogenetic inferences supports a single introduction of the tomato begomovirus into Kenya. There was no evidence of recombination occurring within our ToLCArV population. Intriguingly, five algorithms detected recombination signals (*P* ≤ 0.05) from a Tanzanian ToLCArV isolate (GenBank number DQ519575), identifying one of our Kenyan isolates (GenBank number MN894493) as a major parent (Table 3). This suggests that, although the properties of our isolates are just being characterized, they could be the parents that contributed to the emergence of ToLCArV which was first described by [54]. Thus, it is possible that the Kenyan ToLCV population could pre-date the Tanzanian isolates which were then only reported earlier.

Since our analyses reveal clustering of isolates from geographically proximal countries, the dissemination of the ToLCArV-like isolates is likely to have occurred via virus-infected planting material or spread by cross-border spread of viruliferous whiteflies, leading to genetic similarity among these isolates. Although, our study did not investigate mode of virus transmission, evidence of seed transmission has recently been reported in other closely related begomovirus species from tomato [55] and other hosts [56, 57]. Thus, further research is required to understand how specific begomovirus species are spread across various borders in East Africa and to determine the epidemiological and ecological implications. Additionally, we propose studies to investigate the effect of whitefly-mediated transmission on begomovirus diversity in Kenya.

Interestingly, our results show that the begomovirus sequences from Kenya have discernible patterns of geographical structuring with other ToLCV-like isolates of African origin. This is in agreement with previous studies that have shown geographical structuring of African Old World begomovirus sub-populations into clear genetically distinct categories [58, 59]. This suggests that these viruses perhaps came from a common ancestor that was introduced to the continent and speciation arose as they interacted with various hosts across different geographical locations. In this study, we determined the genetic diversity of ToLCArV-like sequences from Kenyan within tomato fields using coding regions and complete genome sequences. Over the years, tomato begomoviruses in Kenya have received little or no attention in previous studies [60]. Our current findings will deepen the knowledge on genetic diversity of tomato begomoviruses, therefore allowing for better diagnostics and appropriate management options. Our results indicate that although there is low intra-specific diversity among our isolates, the haplotype number and haplotype diversity analyses revealed varying homogenous levels within the coding regions. Thus, the non-coding regions could have contributed to the overall low diversity indices, similar to the observations of [61].

Our results show that varying natural selection pressures appear to be acting on the coding regions of the Kenya ToLCArV-like isolates, indicating independent coevolution of these genes. Our analyses of synonymous and nonsynonymous substitutions revealed that, except the C4 gene, all coding regions appear to be under strong negative or purifying selection to conserve its encoded amino acid sequence. This is in line with similar observations for other related tomato begomovirus species from the Old World [62] and New World [63]. The evolutionary constraints on these coding regions could be intended to preserve their biological functions which include virus replication, accumulation and fidelity to vector transmission. For example, the CP gene has been reported to mediate interactions between begomoviruses and their whitefly vectors [64]. Any alterations in the CP sequence could subsequently alter their virus-vector interactions or other associated biological functions [65]. This is probably why this phenomenon is more in the CP region with the lowest mean dN/dS values, indicating that it is undergoing a stronger purifying selection. Other studies have also indicated similar patterns within begomoviruses, with the CP gene having the strongest evolutionary constraint [66–68]. dN/dS ratios are estimators of evolutionary bottlenecks imposed on a coding region at intra-specific levels. Because natural selection functions largely on these regions, synonymous and nonsynonymous mutations are usually under varying selective pressures and are fixed at different rates within begomovirus genomes [69, 70]. Thus, comparison of synonymous and nonsynonymous substitution rates can reveal the direction and strength of natural selection acting on virus proteins. Importantly, we found the C4 gene within the Kenyan isolates to be selectively neutral as its estimated dN/dS ratio (1.1491) suggests that neither purifying nor diversifying selection was ongoing. This neutral selection could be as a result of its divergent but crucial role in modulating disease severity, determination of host range, virus movement and suppression of host silencing mechanisms [71, 72].

## Conclusions

This study investigated the identity, full sequence properties, genetic diversity, population genetics and presence of recombinants within monopartite begomoviruses associated with leaf curl diseases of tomato in Kenya. Nucleotide and amino acid sequence analyses together with phylogenetic inferences identified the begomoviruses as variants of ToLCArV with origins from Tanzania. Genome analyses revealed low genetic diversity within the population with negative selection occurring within most of the coding regions. The information obtained in this research will assist in the design and implementation of quarantine plans to manage virus-host dynamics. Sequence information and genetic diversity data obtained in this study are also important for the development of rapid and robust detection tools towards the production of virus-free tomato seedlings for farmers. This will ultimately improve tomato production across the country for better food security.

## Supplementary information

**Additional file 1. Fig. S1**: Schematic workflow for taxonomic profiling and virus identification from tomato leaf samples in Kenya.

**Additional file 2. Table S1**: List of tomato begomovirus isolates used across all analyses.

**Additional file 3. Table S2**: Summary of de novo assembly and sequence reads characteristics.

**Additional file 4. Fig. S1**: Schematic workflow for taxonomic profiling and virus identification from tomato leaf samples in Kenya.

**Additional file 5. Table S3**: Percentage nucleic acid similarities between full and individual genomic regions of Tomato leaf curl Arusha virus-like isolates from Kenya with DNA-A component of tomato begomoviruses.

**Additional file 6. Fig. S2**: Pairwise identity of Kenyan monopartite tomato begomoviruses with other tomato-infecting begomovirus species.

**Additional file 7. Table S4**: Percentage amino acid sequence similarities between open reading frames of Tomato leaf curl Arusha virus-like isolates from Kenya with DNA-A component of tomato begomoviruses.

**Additional file 8. File 2**: Aligned begomoviruses used for recombination analyses.

## Data Availability

The datasets supporting the conclusions of this article are available in the NCBI repository at https://www.ncbi.nlm.nih.gov/bioproject/PRJNA646848. All the datasets supporting the findings of this research are included within the article and its additional files. Raw sequence data are accessible at NCBI under the BioSample accession numbers SAMN15566931-SAMN15566941 with SRA accession numbers SRR12245789-SRR12245799. Complete genomes of ToLCArV were deposited to GenBank under Accession Numbers MN894493-MN894502.

## Abbreviations

*Tomato leaf curl*: Arusha virus
ToLCArV: *Tomato leaf curl virus*
ToLCV: *Tomato yellow leaf curl virus*
TYLCV: *Tomato leaf curl Toliara virus*
ToLCToV: ToLCDiV *Tomato leaf curl Diana virus*
ToLCMohV: *Tomato leaf curl Moheli virus*: Deoxyribonucleic acid
DNA: Intergenic region
IR: Open reading frames
ORF: Tris–Hcl
Tris-Hydrochloride: Sodium Chloride
NaCl: Ethylenediaminetetraacetic acid
EDTA: Polyvinylpyrrolidone
PVP: Tagmented DNA Buffer
TD: Tagment DNA Enzyme
TDE: Sequence Demarcation Tool
SDT: Single breakpoint scanning
SBP: Genetic algorithm recombination detection
GARD: Recombination detection program
RDP: Coat protein
CP: Replication gene
Rep: Shortest contig length that covers 50% of the genome
N50: Average nucleotide diversity
π: Haplotype diversity
Hd: Number of polymorphic or segregating sites
S: Number of segregating sites
θ-W: Total number of mutations
Eta: The average number of nucleotide differences between sequences
k: Total number of mutations
θ-Eta: Single-likelihood ancestor counting
SLAC: Hypothesis testing using phylogenies
HyPhy: DNA Sequence Polymorphism
DnaSP: Movement protein
MP: Coat protein
CP: Transcription activator protein
TrAP: Rep enhancer protein
REn: Nonsynonymous substitution per nonsynonymous site
dN: Synonymous substitutions per synonymous sites; dS
