# Supplementary material for: Metagenomic analyses and genetic diversity of Tomato leaf curl Arusha virus affecting tomato plants in Kenya
Source: Virol J. 2021 Jan 6;18:2. doi: 10.1186/s12985-020-01466-z (PMC7789182; doi:10.1186/s12985-020-01466-z)
Supplement: Supplementary file 1 — Additional file 1. Fig. S1: Schematic workflow for taxonomic profiling and virus identification from tomato leaf samples in Kenya. [file 12985_2020_1466_MOESM1_ESM.pdf]

Schematic workflow for taxonomic profiling and virus identification from tomato leaf samples in Kenya

Sample Collection

- Seven counties
- Leaf tissues obtained
- Total of 240 samples
- Pooling and sample selection

Sequencing and Bioinformatics

- Genomic DNA extraction
- Library preparation (Nextera Kits)
- Paired end DNA seqeuncing (Illumina MiSeq)
- Trimming and quality control (FastP)
- Mapping to host reference genome (Bowtie)
- *De novo* assembly of unmapped reads (Megahit)

Virus Identification

- Taxonomic profilling (Kaiju)
- Nucleotide identification (BLASTn)
- Protein prediction (ORF Finder)
- Primer design (Primer Express)
- PCR verification and Sanger sequencing

Distance Matrix and Phylogeny

- Multiple sequence alignment (Clustal-W)
- Pairwise sequence identities (SDT)
- Maximum likelihood tree (MEGA)

Recombination

- Recombination signatures (Datamonkey)
- Recombination signals (RDP)

Population Genetics

- Identification of virus population structure (DnaSP)
- Estimation of selection pressure (HyPhy)
- Test of neutrality (DnaSP)
