# Supplementary figures and images for: Metagenomic analyses and genetic diversity of Tomato leaf curl Arusha virus affecting tomato plants in Kenya
Source: Virol J. 2021 Jan 6;18:2. doi: 10.1186/s12985-020-01466-z (PMC7789182; doi:10.1186/s12985-020-01466-z)

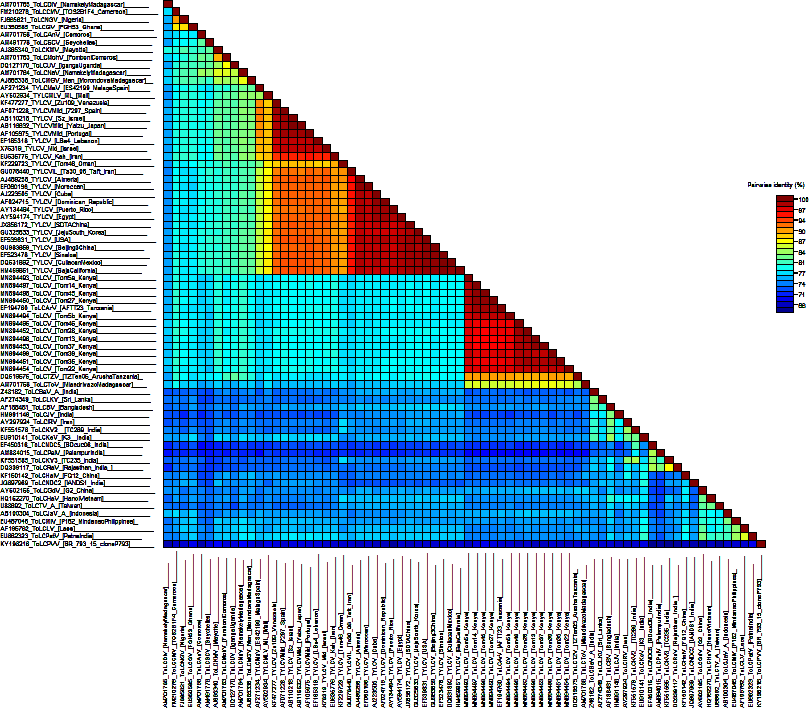

Supplement: Supplementary file 6 — Additional file 6. Fig. S2: Pairwise identity of Kenyan monopartite tomato begomoviruses with other tomato-infecting begomovirus species. [file 12985_2020_1466_MOESM6_ESM.bmp]
